# SEANet: Semantic Enhancement and Amplification for Underwater Object Detection in Complex Visual Scenarios

**DOI:** 10.3390/s25103078

**Published:** 2025-05-13

**Authors:** Ke Yang, Xiao Wang, Wei Wang, Xin Yuan, Xin Xu

**Affiliations:** 1 School of Computer Science and Technology, Wuhan University of Science and Technology, Wuhan 430081, China; yk@wust.edu.cn; 2 Hubei Province Key Laboratory of Intelligent Information Processing and Real-Time Industrial System, Wuhan University of Science and Technology, Wuhan 430081, China; wangwei8@wust.edu.cn (W.W.); xinyuan@wust.edu.cn (X.Y.); xuxin@wust.edu.cn (X.X.)

**Keywords:** underwater object detection, low contrast, feature pyramid network, semantic enhancement

## Abstract

Detecting underwater objects is a complex task due to the inherent challenges of low contrast and intricate backgrounds. The wide range of object scales further complicates detection accuracy. To address these issues, we propose a Semantic Enhancement and Amplification Network (SEANet), a framework designed to enhance underwater object detection in complex visual scenarios. SEANet integrates three core components: the Multi-Scale Detail Amplification Module (MDAM), the Semantic Enhancement Feature Pyramid (SE-FPN), and the Contrast Enhancement Module (CEM). MDAM expands the receptive field across multiple scales, enabling the capture of subtle features that are often masked by background similarities. SE-FPN combines multi-scale features, optimizing feature representation and improving the synthesis of information across layers. CEM incorporates Fore-Background Contrast Attention (FBC) to amplify the contrast between foreground and background objects, thereby improving focus on low-contrast features. These components collectively enhance the network’s ability to effectively identify critical underwater features. Extensive experiments on three distinct underwater object detection datasets demonstrate the efficacy and robustness of SEANet. Specifically, the framework achieves the highest AP (Average Precision) of 67.0% on the RUOD dataset, 53.0% on the URPC2021 dataset, and 71.5% on the DUO dataset.

## 1. Introduction

Underwater object detection (UOD) is a pivotal research domain underpinning a variety of visual tasks, including underwater biological tracking [1,2], underwater image classification [3], and seabed modeling [4]. Recently, the exploration of complex underwater environments has increasingly relied on autonomous underwater vehicles, remotely operated vehicles, and other advanced robots, which have supplanted traditional human labor. These robotic systems form an integrated underwater visual perception network for identifying marine organisms by analyzing video footage, images, and echo signals reflected from objects. By accurately identifying these marine organisms, the system facilitates the rapid, automatic acquisition of biological data, making it an essential prerequisite for further underwater exploration. However, the behavior and morphological characteristics of aquatic organisms, along with the distinct conditions of underwater environments, present significant challenges for underwater exploration. Captured images often exhibit low contrast between objects and their backgrounds, primarily caused by color distortion and the inherent complexity of the underwater environment. Additionally, marine organisms manifest at multiple scales, with many objects particularly susceptible to low contrasts, which reduces detection accuracy. We define this as Low-Contrast and Multi-Scale Underwater Object Detection (LMUOD).

Specifically, underwater object detection presents unique characteristics that distinguish it from general object detection tasks, mainly because of the uniqueness of the underwater environment. **(1) Image distortion**: Underwater images often experience color shifts and fogging due to the water’s absorption and scattering effects. Research efforts [5,6,7,8,9] have focused on investigating the connection between underwater image enhancement (UIE) and object detection to address these issues. The approaches generally fall into two categories: preprocessing and multi-task learning. In preprocessing, UIE is treated as an initial step before object detection. In contrast, multi-task learning involves training both enhancement and detection models simultaneously to improve detection through enhanced images. UIE typically requires raw images and enhanced images during training, but obtaining these paired images is highly challenging in real-world underwater environments. **(2) Dense and crowded targets**: Aquatic organisms often inhabit crowded environments in groups, leading to occlusion and overlapping objects. It makes object detection particularly challenging. To address these issues, some researchers have developed innovative methods. For instance, Shi et al. [10] utilized SIOU-Softnms to handle commonly missed detections in dense and small object scenarios by removing redundant object boxes during post-processing. They also designed a Locally Enhanced Position Encoding Attention Module to capture features of small objects efficiently. Similarly, Qi et al. [11] proposed a pyramid structure incorporating multiple deformable convolutional layers to tackle occlusion and object deformation challenges. **(3) Dataset limitations**: Creating high-quality underwater datasets is tough due to the need for specialized equipment and techniques, resulting in high data collection costs and limited dataset sizes. In addition to the development of new datasets, many efforts have focused on data augmentation to mitigate dataset limitations and enrich the target domain data. Despite some progress in addressing the aforementioned issues, challenges persist in underwater scenarios characterized by significantly low contrast and scale variation. This emphasizes the urgent need for ongoing and thorough research and development in underwater object detection.

**Figure 1:**
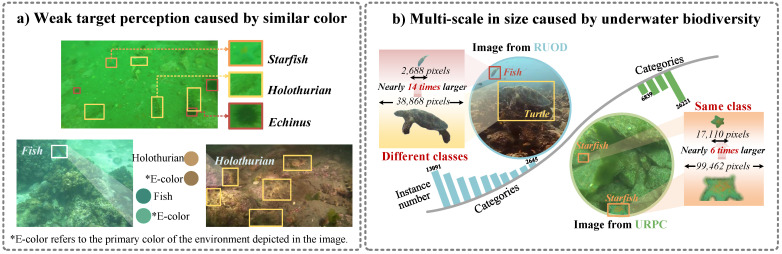
**The challenges of underwater object detection.** (**a**) The underwater environment often features low contrasts between objects and their backgrounds, leading to weak target perception. The lack of contrast makes it hard for detection models to distinguish weak targets, hindering accurate identification and localization. (**b**) The biodiversity of underwater organisms leads to significant scale differences, where the bounding boxes of larger objects may be several times larger than those of smaller objects. This disparity presents substantial challenges for object detection.

LMUOD is particularly challenging due to two significant obstacles. **Challenge 1: weak target perception:** Many underwater organisms utilize camouflage to blend seamlessly into their environments, making it difficult to distinguish them from their surroundings. As depicted in Figure 1a, some organisms nearly vanish against their backgrounds due to their protective coloration, which closely resembles their habitats. This characteristic presents a challenge for models to extract critical features for accurate detection, particularly in low-contrast environments. **Challenge 2: multi-scale size variability.** Underwater organisms exhibit significant size variability. Additionally, variations in camera angles and the distance between the organism and camera further exacerbate size differences between objects. As shown in Figure 1b, there are substantial size disparities both between different species and within the same species. For example, the bounding box for the turtle covers 17.4% of the image, while that for the fish occupies only 1.2%. This necessitates the model’s ability to extract features from various scales. Both challenges significantly increase the difficulty of object detection in complex visual scenarios, underscoring the need for advanced and robust methodologies that can effectively address the issues and improve detection accuracy.

To tackle the challenges in LMUOD, we propose a specialized framework named the Semantic Enhancement and Amplification Network (SEANet). The source code is available at SEANet (https://github.com/Nicoleyk/SEANet, accessed on 1 August 2024). SEANet improves the model’s capacity to identify and locate targets in challenging underwater environments by amplifying and enhancing important semantic information in the image. Inspired by the principles of human visual perception, SEANet addresses the issue where, in complex scenes, objects often exhibit low contrast with their surroundings, leading to blurred boundaries. The human eye adapts to such conditions by adjusting its focal point and viewing angle to accommodate objects at various distances and scales. Additionally, it distinguishes objects by comparing brightness differences between the foreground and background, with higher contrasts making the foreground more prominent. Building on these principles, SEANet incorporates specific design elements. **(1) Multi-scale detail amplification module (MDAM)**: Recognizing the difficulty that general feature extraction architectures face in complex underwater environments due to the similar texture structures between organisms and their surroundings, we develop the MDAM. This module enhances the extraction of refined multi-scale features by capturing subtle cues that are often overlooked. **(2) Semantic enhancement feature pyramid (SE-FPN)**: To optimize the integration of features at different scales, we develop a feature pyramid structure tailored for underwater contexts. This structure enables the effective use of feature information across various channels in the feature maps, thus enhancing the model’s capacity to handle and combine data from multiple scales. **(3) Contrast enhancement module (CEM)**: We incorporate Fore-Background Contrast Attention (FBC) to construct the CEM within the pyramid structure, increasing the contrast between foregrounds and backgrounds. FBC is designed to interpret the learned features by distinguishing between two key elements: biological traits influenced by environmental factors, which are harder to detect, and irrelevant background details. The FBC enhances the semantic focusing ability by effectively distinguishing these elements, thereby improving the model’s attention to low-contrast targets. Together, these innovations in SEANet aim to overcome the inherent challenges of detecting low-contrast and multi-scale objects in underwater environments, drawing on biological insights to inform advanced computational techniques.

In summary, the major contributions of this paper are as follows:

- We proposed a specialized framework called the Semantic Enhancement and Amplification Network (SEANet) to address the issues in LMUOD. SEANet, inspired by human visual perception principles, enhances the model’s ability to recognize and localize targets in challenging underwater conditions by amplifying image semantic information.
- We developed the Multi-Scale Detail Amplification Module (MDAM) to address the challenges faced by general feature extraction architectures in complex underwater environments due to similar texture structures. MDAM captures subtle cues that are often overlooked, improving the extraction of detailed multi-scale features.
- We developed the Semantic Enhancement Feature Pyramid (SE-FPN) to optimize multi-scale feature integration in underwater contexts, enhancing the model’s ability to process and integrate multi-scale data. Additionally, the Contrast Enhancement Module (CEM) within the pyramid structure introduces the Fore-Background Contrast Attention (FBC) mechanism. In the FBC, ambiguous features are interpreted as a combination of different information types, and differentiating them enhances the semantic focusing ability, thereby improving the model’s focus on low-contrast objects.
- SEANet achieves state-of-the-art performance, with the highest Average Precision (AP) recorded at 67.0% on the RUOD dataset, 53.0% on URPC2021, and 71.5% on the DUO dataset, demonstrating its effectiveness and robustness in addressing the challenges of underwater object detection.

## 2. Related Works

In this section, we begin by presenting an overview of some representative approaches to underwater object detection. Through a review of the literature, we categorize them into two-stage detectors, one-stage detectors, and transformer-based detectors. We then provide a brief summary of the feature pyramid.

### 2.1. Underwater Object Detection

#### 2.1.1. Two-Stage Object Detector

Lv et al. [12] employed a foreground–background segmentation weak fitting network in the first stage and a refinement network in the second stage. Traditional U-Net architectures perform poorly in these scenarios. Consequently, they designed a lightweight U-Net to minimize the model’s overfitting to the training dataset, directing the creation of positive and negative samples based on segmentation outcomes. Lin et al. [13] introduced a data augmentation method that blends proposals from various images, effectively simulating scenarios involving overlapping, occlusion, and blurring of objects. This approach is applied between the Region Proposal Network (RPN) and the Region of Interest (RoI), where randomly generated proposals from the RPN are combined to create new proposals. Song et al. [14] built on the R-CNN framework and proposed a two-stage detector that incorporates uncertainty modeling and hard sample mining. They first measure the ambiguity of objects through IoU predictions. A probabilistic inference pipeline uses the uncertainty from the initial stage to recalibrate weights, allowing the subsequent detector to focus more on difficult samples that were previously miscalculated. Due to the adverse underwater environment altering the frequency content of features, Pang et al. [15] approached the problem from a frequency perspective, extracting irrelevant content from frequency features. They optimized a magnitude corrector to gradually align the frequency content between high-quality images and noisy images, mitigating noise-induced environmental interference. However, underwater object detection approaches using a two-stage process generally have higher model complexity and lower detection efficiency.

#### 2.1.2. One-Stage Object Detector

Hu et al. [16] proposed a multi-directional edge detection algorithm that enhances edge features by leveraging the shape characteristics of sea urchins. Fan et al. [17] refined multi-scale features to better align them with anchor points by performing regression during preprocessing to remove obvious backgrounds. The results from this stage were then used to compute offsets via DCN, leading to finer localization and classification outcomes. Cai et al. [18] introduced a weakly supervised learning strategy, allowing two detectors to select cleaner samples for mutual teaching and alleviating noise through batch-filtered samples. Zhou et al. [19] incorporated channel attention into the direct edge mapping of residual structures, adding shallow feature information to deep networks, and combined hard sample resampling with focal loss to increase the contribution of minority classes to the total loss during backpropagation. Ouyang et al. [20] designed a lightweight underwater object detector that leverages the advantages of convolutional neural networks and vision transformers for learning global representations, significantly reducing parameters with a novel upsampling method based on semantic alignment. Chen et al. [21] integrated DCNv3 into the basic blocks of the backbone network, introducing an integrated feature fusion framework that leverages channel, scale, and spatial attention. Zhou et al. [22] aimed to mitigate underwater noise impacts by introducing vortex convolutions to disrupt noise distributions while designing a module that enhances long-range and short-range feature associations, thereby improving network performance in complex underwater environments. Despite advances in underwater object detection, these methods still struggle with challenges in effectively addressing low-contrast issues, highlighting the urgent need for improvements in detection accuracy.

#### 2.1.3. Transformer-Based Object Detector

Gao et al. [23] utilized the CSWin transformer as a baseline and proposed a path-enhanced transformer framework that integrates localized path detection data to enable communication between high-level and low-level features, aiming to address the challenges of detecting small underwater objects. Additionally, Wang et al. [24] introduced an improved transformer for dynamic fish detection, taking into account the surface patterns of fish bodies and their deformations while swimming. The backbone network incorporates deformable convolutions, and the Slim Hybrid Encoder serves as the neck component, designed to integrate fish body feature information and tackle the issue of body deformation during movement. Shah et al. [25] integrated transformer modules into the YOLOv8 framework to improve the perception of contextual semantics and global information. Transformer models typically require longer computation times and substantial memory. As a result, these methods are relatively rare in underwater object detection.

### 2.2. Feature Pyramids

The multi-scale feature pyramid combines information from various levels, playing a crucial role in object detection. Initially introduced by Lin et al. [26], the FPN architecture has become a widely adopted and extensively studied approach. PANet [27] introduces an additional bottom–up pathway to the FPN structure to enhance the details of deep features. Pang et al. [28] propose that feature fusion should incorporate balanced information from various scales. They introduce the concept of balance, averaging features from different scales to create balanced features. BiFPN [29] introduces a bidirectional cross-scale pathway. SA-FPN [30] utilizes a top–down upsampling path to refine each feature map with information from higher levels. This feature pyramid is designed to extract detailed and robust features, thereby improving the accuracy of marine object detection. Qiao et al. [31] argue that the performance of small object detection is challenged by varying feature scales and overlapping representations. To mitigate the impact of feature aliasing, they introduce UEFPN, which utilizes a unified feature domain. Gao et al. [32] propose an enhanced weighted bidirectional feature pyramid network AWBiFPN to counteract the degradation of underwater image features and enhance the efficiency of multi-scale feature fusion. However, these feature pyramids are not specifically designed for low-contrast scenarios.

## 3. Method

**Figure 2:**
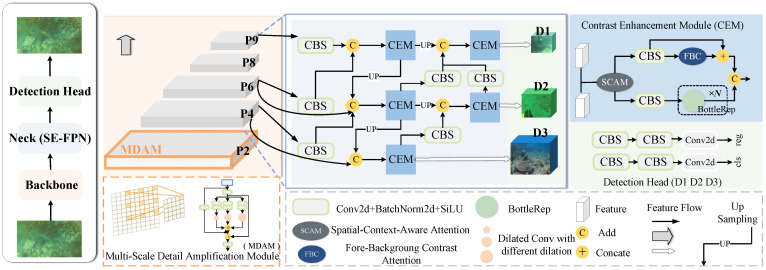
**Overall architecture of SEANet.** The whole detection model is divided into three parts: backbone, neck, and detection head. P2-P9 are the feature extraction layers of the network. D1, D2, and D3 refer to three detection heads. SE-FPN is the feature pyramid that we proposed.

Our objective is to enhance feature learning in Low-Contrast and Multi-Scale Underwater Object Detection (LMUOD), enabling the more effective recognition of targets in low-contrast environments while reducing background interference. Additionally, we aim to fully integrate multi-channel information to address the challenges of multi-scale detection and limited perceptual capabilities. To achieve this, we perform multi-scale detail amplification in the shallow layers of the network to enhance fine-grained target details. Furthermore, we introduce a Low-Contrast Stage to design a new semantic enhancement feature pyramid, which amplifies the contrast between the target and background. This improves the model’s ability to prioritize the semantic information of the target. The overall architecture of the model is presented in Figure 2.

### 3.1. Enhanced Feature Extraction with MDAM

**Figure 3:**
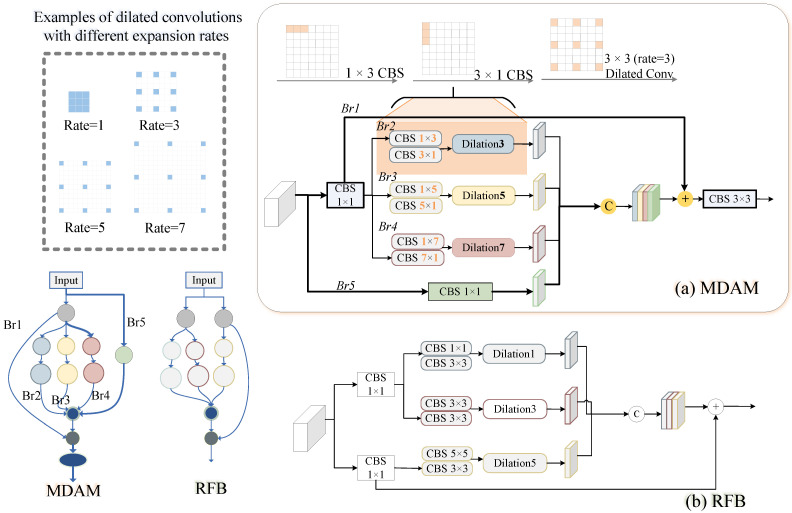
**Demonstration of submodules.** The architecture of our Multi-Scale Detail Amplification Module (MDAM) and the foundation work of RFB [33]. MDAM includes five distinct branches, each equipped with different kernels, which enhance the ability to capture discriminative contexts at multiple scales. The five branches are denoted as Br1 to Br5.

Feature extraction is a fundamental and crucial component of object detection. The initial layers of the network are responsible for extracting detailed features closely related to the input image, which are essential for accurate object detection. However, the similarity in texture between underwater objects and their surrounding environments diminishes the model’s ability to detect these objects, leading to a reduction in important information during the extraction process. Generic feature extraction structures often struggle to capture these subtle details accurately. To address this challenge, we propose a feature extraction network aimed at enhancing target information extraction in complex backgrounds. Drawing inspiration from the human visual perception system, we expand the receptive field to capture higher-resolution contextual data. Our Multi-Scale Detail Amplification Module (MDAM) is introduced at the second layer of the backbone network. As shown in Figure 3a, MDAM adopts a multi-branch structure, combining adaptive convolutional kernels and receptive field mechanisms, with convolutional kernels of different sizes designed to explore texture and detail information at various scales locally.

MDAM consists of five branches. The first convolutional layer (CBS) in four branches has a size of 1×1 for dimensionality reduction. After the 1×1 CBS operation, branches *i* use $k_{i}\times 1$ and $1\times k_{i}$ convolution layers, reducing model parameters without compromising performance. Next, dilated convolutions with dilation rates of $k_{i}$ are used to expand the receptive field. Here, $k_{i}=2\times i-1$, with *i* representing the branch index (i=2,3,4). This value determines the size of the convolution kernels used in each branch, with the kernel size increasing as the branch index increases. The fifth branch applies a 1×1 CBS operation directly to the input. Branch j (where j = 2, 3, 4, 5) is concatenated and then added to branch 1, resulting in a final output that is subsequently fed into a 3×3 CBS. The CBS block consists of a convolution layer, group normalization, and an ReLU activation. MDAM replicates the behavior of receptive field areas in human vision, improving the extraction and emphasis of essential features from the input. The whole process is expressed as follows:

(1) $$X_{\mathrm{out}}=\mathrm{Conv}\left(Br_{1}⊕\mathrm{concat}\left(Br_{2},Br_{3},Br_{4},Br_{5}\right)\right)$$

where $X_{out}$ represents the output feature; $Br_{1}$, $Br_{2}$, $Br_{3}$, $Br_{4}$, and $Br_{5}$ indicate the outputs generated by each of the five branches. concat(·) denotes the feature concatenation operation, ⊕ denotes the feature addition operation, and Conv(·) is the final CBS operation before the output of the module. Assuming *x* is the input feature, $x^{\prime }$ is the feature after the first 1×1 CBS, and Equation (1) can be written in detail as follows:

(2) $$X_{\mathrm{out}}=\mathrm{Conv}\left(x^{\prime }⊕\mathrm{concat}\left(\delta_{2}\left(\epsilon_{2}\left(x^{\prime }\right)\right),\;\delta_{3}\left(\epsilon_{3}\left(x^{\prime }\right)\right),\;\delta_{4}\left(\epsilon_{4}\left(x^{\prime }\right)\right),\;\epsilon_{5}\left(x\right)\right)\right)$$

Here, $\epsilon_{5}$ refers to the convolution operation in the fifth branch $Br_{5}$. $\epsilon_{i}$ represents the convolution layers of (2i−1)×1 and 1×(2i−1) in $Br_{i}$, and $\delta_{i}$ denotes the operation of receptive field expansion in $Br_{i}\left(2\leq i\leq 4\right)$.

Compared to RFB [33], MDAM offers a more diverse multi-scale receptive field strategy, encouraging the network to capture discriminative features through an asymmetric design in underwater scenarios. Unlike RFB, which directly activates the features, MDAM achieves both secondary extraction and normalization by incorporating a CBS block before the final output. The integration of CBS blocks before the final output aids in regularizing the feature maps, improving training stability and enhancing model robustness in challenging environments. Moreover, branch 5 retains the original local details, ensuring that fine-grained information is maintained and effectively incorporated into feature fusion, which is crucial for preserving spatial consistency in underwater object detection. MDAM optimizes feature processing in a more refined manner, enabling the model to perform more robustly in diverse environments.

### 3.2. Advanced Fusion for Low-Contrast Features

#### 3.2.1. Revisiting FPN

**Figure 4:**
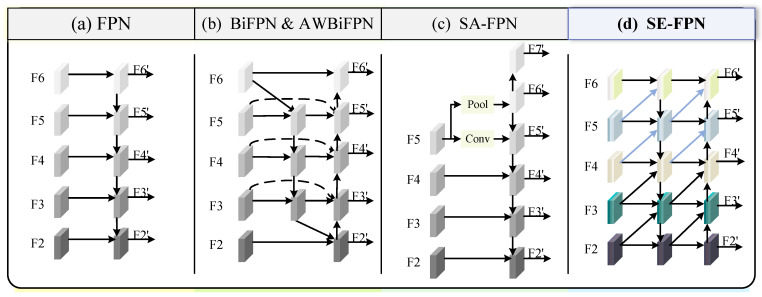
**Comparison of various feature pyramids.** {F2,F3,F4,F5,F6,F7} represents a set of features extracted at various scales. $\left\{F2^{\prime },F3^{\prime },F4^{\prime },F5^{\prime },F6^{\prime },F7^{\prime }\right\}$ is the set of features generated by the constructed feature pyramid. (**a**) FPN introduces a top-to-bottom pathway; (**b**) BiFPN and AWBiFPN have the same fusion pathway, but AWBiFPN replaces DWConv in BiFPN with ordinary conv; (**c**) SA-FPN proposes a scale-aware feature pyramid; (**d**) illustrates the feature fusion concept of our SE-FPN, where features from the current layer are concatenated with features from previous layers at different scales using a simple concatenation operation, without applying any weighting or complex fusion strategy.

The Feature Pyramid Network is commonly employed in object detection. Multi-scale feature fusion refers to combining features from various scales based on specific rules. Figure 4 illustrates several feature fusion methods of feature pyramids, where FPN [26] and BiFPN [29] are designed for general object detection, while AWBiFPN [32] and SA-FPN [30] are specifically developed for underwater object detection. For the basic FPN, a top-to-bottom pathway is used to integrate features across multiple scales, ranging from level 2 to 6 (F2−F6). The features are fused from the top to the bottom, as shown in Figure 4a. The feature fusion path of BiFPN and AWBiFPN is illustrated in Figure 4b. BiFPN introduces cross-layer connections in addition to bidirectional transmission. Additionally, it assigns more weight to each input, allowing the network to prioritize the relevance of individual features during learning. The convolution used in BiFPN is depthwise separable convolution (DwConv). Since DwConv processes each channel independently and lacks cross-channel feature fusion, it is limited in capturing inter-channel dependencies and complex features. AWBiFPN replaces DwConv in BiFPN with ordinary convolution to improve accuracy. The construction of other features in AWBiFPN follows the same methodology as in BiFPN. SA-FPN in Figure 4c proposes a feature pyramid framework that adapts to different scales to extract abundant robust features from underwater visuals. SA-FPN takes FPN as a baseline. Compared with FPN, $F5^{\prime }$ in SA-FPN is extracted from F5 with the convolutional operation, while $F6^{\prime }$ is downsampled from F5 by max pooling. SA-FPN further introduces a higher-level feature map $F7^{\prime }$ to obtain additional context information. Figure 4d shows the structure of our Semantic Enhancement Feature Pyramid (SE-FPN). In SE-FPN, multi-scale features are fused in the previous and current layer levels. To maintain consistency in the description, we illustrate the input feature list of SE-FPN as consisting of five layers. In reality, however, in our SEANet architecture, only three output features from the backbone are fed into the SE-FPN.

#### 3.2.2. Semantic Enhancement Feature Pyramid

**Figure 5:**
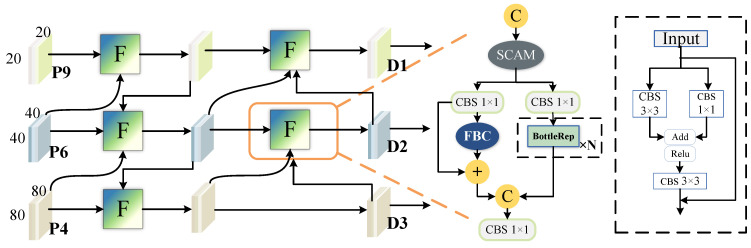
**The detailed structure of SE-FPN.** *F* represents the Contrast Enhancement Module (CEM). CBS combines a convolution layer, a batch normalization layer, and the SiLU activation function. P4, P6, and P9 are three outputs from the 4th, 6th, and 9th layers of the feature extraction. D1, D2, and D3 represent the three outputs produced by the Neck network. Finally, D1, D2, and D3 are sent to the detection head for prediction.

Although AWBiFPN and SA-FPN enhance multi-scale feature fusion and improve object detection performance to some extent, they do not specifically address the problem of low contrast between underwater organisms and their surrounding environment, resulting in weak perception capability of the model for underwater targets. To address this issue, we designed SE-FPN, which aims to enhance the model’s attention to and differentiation of semantic information in underwater object detection through multi-scale feature fusion and contrast enhancement between the foreground and background. Specifically, “semantic enhancement” refers to the effective fusion of features from different scales to help the model better understand and distinguish target features in underwater environments, especially in low-contrast backgrounds. In this way, SE-FPN not only strengthens the model’s ability to perceive targets but also improves its ability to accurately recognize target semantic information in complex underwater environments. As shown in Figure 4d, for each level of SE-FPN, in addition to the feature map of the current layer, feature maps from the previous layer at different scales are directly added to the input of the current layer. Shallow features contain rich local details, while deep features primarily capture semantic information. Transferring shallow features to higher-level features helps preserve detailed information and facilitates the effective transmission of contextual information. The feature fusion enhances the model’s ability to recognize multi-scale objects, thereby improving its performance in handling complex underwater scenes. Figure 5 shows the detailed structure of our SE-FPN, with the network primarily composed of the Contrast Enhancement Module (CEM).

**Figure 6:**
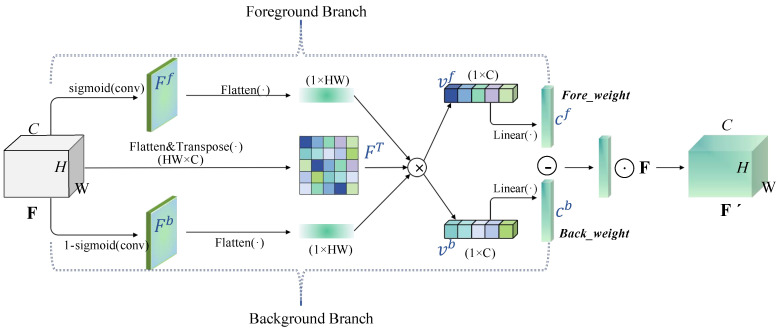
**Illustration of FBC.**⊗: Matrix multiplication; ⊖: vector difference; ⊙:element-wise product.

We first use convolution operations or upsampling to align features from different spatial scales to a consistent dimension and then input them into the CEM module for further processing. The operation of CEM is detailed in Algorithm 1. SCAM is a simple spatial-channel-aware attention mechanism [34]. It first applies channel attention to weight the input features and then applies spatial attention, finally combining the weighted results from both attention mechanisms. After passing through SCAM, the features are divided into two branches. One branch performs the BottleRep operation three times, which is a commonly used basic residual block in the YOLO series. The other branch incorporates the Fore-Background Contrast Attention (FBC). The structure of FBC is illustrated in Figure 6. FBC treats features as two categories: one for the target features and another for background information. It focuses on contrastive learning by calculating the vector differences that distinguish underwater organisms from their surrounding environment.
**Algorithm 1** Forward Pass of CEM**Input:** Feature map *x* with shape [batch_size,channel,height,width]
**Output:** Feature map *y* with shape [batch_size,channel_out,height_out,width_out]
1:x←SCAM(x)2:$y_{1}\leftarrow CBS_{1}\left(x\right)$3:$y_{1}\leftarrow FBC\left(y_{1}\right)+y_{1}$4:$y_{2}\leftarrow CBS_{2}\left(x\right)$5:$mid\_out\leftarrow [y_{1}]$6:**for** each convolutional_block in convs **do**7:    $y_{2}\leftarrow \mathrm{error}\left(y_{2}\right)$ \\convolutional_block refers to BottleRep8:    $mid\_out.\mathrm{append}(y_{2})$9:**end for**10:$y\leftarrow CBS_{3}\left(\mathrm{Concatenate}\left(mid\_out,\mathrm{axis}=1\right)\right)$11:**return** *y*


Specifically, for a given underwater image, the feature layer input is $F=[f_{1},f_{2},\ldots ,f_{C}]$, where *C* represents the number of channels. We first use sigmoid activation on the feature map, which has been compressed into a one-dimensional form, to decompose the attention allocated to low-contrast target features from that assigned to the background information. The corresponding equations for the underwater target activation map $F^{f}$ and the background information activation map $F^{b}$ are as follows:

(3) $$F^{f}=\delta \left(CBLR\left(F\right)\right),$$

(4) $$F^{b}=1-F^{f},$$

where CBLR(·) means the Conv2d,BatchNorm2d, and LeakyRelu functions, and δ denotes the sigmoid function.

Following the previous step, the shapes of $F^{f}$ and $F^{b}$ are both [B,1,H,W]. The feature map *F* is then divided into two components for underwater organisms and the background using the low-contrast activation maps: One part highlights the underwater object features, while the other represents the background information. These two components are denoted as $v^{f}$ and $v^{b}$, respectively, and can be formulated as follows:

(5) $$v^{f}=F^{f}⊗F^{T},$$

(6) $$v^{b}=F^{b}⊗F^{T},$$

where the symbol ⊗ represents matrix multiplication, and *T* denotes matrix transposition. We flatten $F^{f}$, $F^{b}$, and $F^{T}$, changing their shapes to [B,1,H×W], [B,1,H×W], and [B,H×W,C], respectively. After the ⊗ operation, the resulting shapes of $v^{f}$ and $v^{b}$ are [B, 1, C].

The operations mentioned above integrate the features of underwater organisms with background information, implementing dimension reduction and feature decomposition to enhance the differentiation between foreground and background information in low-contrast scenarios. Through sigmoid activation mapping, low-contrast feature representations for underwater organisms and background features are generated. This aids subsequent networks in capturing the long-term dependencies between low-contrast target regions and background areas, thereby facilitating more effective target recognition in complex underwater environments.

To leverage both feature representations, we introduce a simple gating mechanism with a sigmoid activation function. This allows for the optimal utilization of the captured information from both organisms and the background, thereby improving the differentiation between them.

(7) $$c^{f}=\delta \left(\rho \left(v^{f}\right)\right),$$

(8) $$c^{b}=\delta \left(\rho \left(v^{b}\right)\right),$$

where ρ denotes the linear function, and $c^{f}$ and $c^{b}$ refer to the feature channel for low-contrast target and background information, respectively. We compute the vector difference between $c^{f}$ and $c^{b}$ to enhance the contrast between the two feature types. The resulting low-contrast underwater object features, after adaptive adjustment, are given by the following:

(9) $$F^{\prime }=F\cdot \left(c^{f}-c^{b}\right).$$

### 3.3. Detection Head

The three outputs from our SE-FPN are passed through the detection head for final predictions. We adopt the original detection head from the baseline gelans [35] and retain its default loss function configuration. Specifically, the category classification loss uses Binary Cross Entropy (BCE) Loss, while the regression loss employs Distribution Focal Loss (DFL) and Complete Intersection over Union (CIoU) Loss. The total loss is computed by weighting these three components according to predefined proportions, which can be expressed as follows:

(10) $$target\_scores\_sum=max(\sum_{i=1}^{N}{target\_scores}_{i},1),$$

(11) $$box\_loss=\frac{\sum_{i=1}^{N}\left(1-CIoU\left(pbox_{i},tbox_{i}\right)\right)\cdot box\_weight_{i}}{target\_scores\_sum},$$

(12) $$cls\_loss=\sum_{i=1}^{N}BCE\left(pscore_{i},tscore_{i}\right),$$

(13) $$dfl\_loss=\frac{\sum_{i=1}^{N}DFL\left(pbox_{i},tbox_{i}\right)\cdot box\_weight_{i}}{target\_scores\_sum},$$

(14) TotalLoss=box_loss×7.5+cls_loss×0.5+dfl_loss×1.5,

where ${target\_scores}_{i}$ denotes the confidence score of each predicted bounding box, typically ranging from [0, 1], with 0 indicating the absence of a target and 1 indicating complete certainty of its presence. *N* represents the number of matched boxes. $box\_weight_{i}$ is the weight assigned to each predicted bounding box based on its confidence level. $pbox_{i}$ and $tbox_{i}$ refer to the $i_{th}$ predicted and target bounding boxes, respectively, while $pscore_{i}$ and $tscore_{i}$ denote their corresponding confidence values. CIoU(·), BCE(·), and DFL(·) refer to the commonly used CIoU Loss, BCE Loss, and DFL Loss, respectively.

## 4. Experiments

In this section, we conduct a series of experiments to evaluate the performance of our proposed method and analyze the results. We first describe the three datasets used in our experiments, followed by an outline of the implementation details and evaluation metrics. Next, we compare our approach with other detectors through experiments on the datasets and provide a detailed ablation study to assess the impact of each component. Finally, we provide visualizations of some of the results and analyze the robustness of our detector in various challenging scenarios.

### 4.1. Datasets

**RUOD** [36] is a large real-world underwater object detection dataset. Data collection for RUOD lasted over a year and was not conducted in a specific scenario. Instead, it was designed for general underwater detection, covering a variety of challenges such as light distortion, haze effects, and various intricate marine conditions. The dataset features a large volume of data with high-quality annotations, it consists of two non-crossing subsets (a training set and a validation set), which contain 9800 and 4200 images, respectively. The dataset contains a total of 74,903 annotated objects, spanning 10 underwater object categories, including fish, sea urchins, corals, starfish, sea cucumbers, scallops, divers, squid, sea turtles, and jellyfish. Object heights range from 1 pixel to 3618 pixels, and the distribution of objects across categories is uneven. Fish account for approximately 17.5%, sea urchins for 15.1%, and corals for 11.9%, with the remaining categories comprising smaller proportions. Each image typically contains between 1 and 15 objects, with an average of 9.57 object instances per image.

**URPC2021** (http://2021en.urpc.org.cn/index.html (accessed on 1 August 2024)): This dataset is released in the 2021 China Underwater Robot Professional Contest, which contains four categories: holothurian, echinus, scallop, and starfish. All images are captured near Zhangzi Island, Dalian, China. The competition offers a total of 7600 images along with their associated annotation data. We randomly divide all images in a 9:1 ratio, resulting in a training set of 6080 images and a validation set of 1520 images. The training and validation sets are entirely separate, with no overlap between them, ensuring their independence.

**DUO** [37] contains 7782 images after deleting overly similar images, with 6671 for training and 1111 for testing. The total number of objects is 74,515. The dataset includes a total of 74,515 annotated objects, with 7887 sea cucumbers, 50,156 sea urchins, 1924 scallops, and 14,548 starfish. Most objects occupy between 0.3% and 1.5% of the image area. Sea urchins are the most abundant, comprising 67.3% of the total objects. The number of objects per image typically ranges from 5 to 15, with an average of 9.57 object instances per image. Additionally, due to the small size of the objects and the high image resolution, the DUO dataset exhibits a significant long-tail distribution.

### 4.2. Implementation Details and Evaluation Metrics

**Experimental details:** Before training, all images are resized to 640 × 640 pixels. We only apply the default data augmentation technique, Mosaic, which is disabled during the last 10 epochs to allow the model to be fine-tuned with a more realistic data distribution. The training process runs for 300 epochs on a compute node with two RTX 3090 GPUs, each with 24 GB of memory, and early stopping is employed. Our method does not utilize any pre-trained models. The parameters used to analyze the experiments in this paper are shown in Table 1.

**Evaluation metrics:** We adapt AP, $AP_{50}$, and $AP_{75}$ as the primary metrics for model accuracy evaluation, with *Precision (P)*, *Recall (R)*, and *F*1 *score*(*F*1) as supplementary indicators. AP is the average precision computed across multiple IoU thresholds, from 0.5 to 0.95 with a step size of 0.05. $AP_{50}$ refers to the average precision at an IoU threshold of 0.5, indicating a less strict requirement for detection, while $AP_{75}$ reflects the average precision at a higher IoU threshold of 0.75, requiring more precise matches. Furthermore, we include the number of parameters as an additional metric to assess the size of different models. *P*, *R*, and *F*1 are expressed as follows:

(15) P=TP/(TP+FP),

(16) R=TP/(TP+FN),

(17) F1=2×P×R/(P+R),

where TP and FP refer to correct and incorrect predictions for positive examples, respectively, while TN and FN represent correct and incorrect predictions for negative examples.

### 4.3. Comparisons with Other Detectors

To evaluate the effectiveness of our method, we compare SEANet with various methods across three underwater datasets. The selected representative methods include both generic object detection methods and those specifically designed for underwater object detection. The experimental results clearly indicate that our method outperforms the others, achieving better performance in underwater object detection.

**Figure 7:**
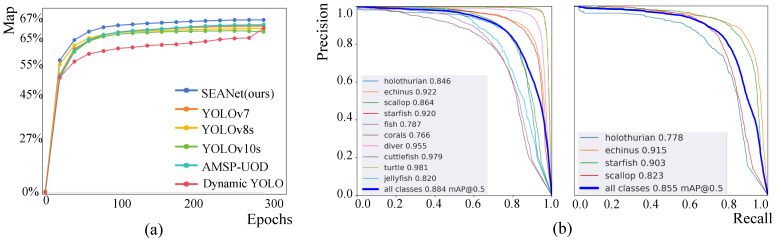
(**a**) presents the AP growth curve during training on the RUOD validation dataset for object detection algorithms. (**b**) represents the P-R curve of SEANet on the RUOD and URPC2021 validation datasets. It demonstrates the $AP_{50}$ of our SEANet for each category on both datasets.

*(1) Results on RUOD:* The experiment results on the RUOD dataset are shown in Table 2. Our SEANet performs better on the three primary metrics (AP, $AP_{50}$, and $AP_{75}$), as well as the supplementary metrics. It surpasses other detectors, setting a new benchmark for state-of-the-art performance, as illustrated in Figure 7a. SEANet achieves 67.0% AP, 88.4%$AP_{50}$, 73.9%$AP_{75}$, 82.0% R, 87.6% P, and 84.7% F1. SEANet outperforms the Detectors [38] by 9.2% (57.8% vs. 67.0%), YOLOv7 [39] by 2.4% (64.6% vs. 67.0%), Dynamic YOLO [21] by 3.3% (63.7% vs. 67.0%), and GCC-Net [8] by 7.6% (59.4% vs. 67.0%) on AP. Among the methods, AMSP-UOD [22] achieves the second highest values on AP and $AP_{75}$, YOLOv7 attains the second highest values on $AP_{50}$ and F1, and YOLOv10 [40] achieves the second highest values on P. SEANet outperforms the current highest benchmarks by 1.8% (67.0% vs. 65.2%) on AP, 0.4% (88.4% vs. 88.0%) on $AP_{50}$, and 1.4% (73.9% vs. 72.5%) on $AP_{75}$.

*(2) Results on URPC2021:* The experiment results on the URPC2021 dataset are shown in Table 3. SEANet achieves 53.0% AP, 85.5% $AP_{50}$, and 60.3% $AP_{75}$, achieving the highest performance. In terms of AP metrics, our SEANet achieves a detection accuracy of 85.5%, outperforming other models. This represents an improvement of 3.3% over YOLOv7, 1.8% over YOLOv10m, and 0.3% over Dynamic YOLO. For the $AP_{50}$ metric, SEANet achieves 85.5%, which is the highest detection accuracy among all models, surpassing GCCNet by 1.7% and YOLOv10m by 0.7% and being the same as Dynamic YOLO. As for the $AP_{75}$ metric, SEANet reaches the highest detection accuracy at 60.3%, surpassing all other methods.

*(3) Results on DUO:* As shown in Table 4, SEANet achieves 71.5% AP, 87.8% $AP_{50}$, and 79.1% $AP_{75}$ on the DUO dataset. In terms of category-wise AP, the network achieves 71.4%, 77.6%, 57.8%, and 79.0% for holothurian, echinus, scallop, and starfish, respectively. While certain detectors may excel in specific categories, SEANet delivers more consistent results across all target classes.

### 4.4. Ablation Study

In this section, we evaluate the impact of different components in our proposed SEANet model on the RUOD and DUO datasets. As shown in Table 5 and Table 6, substituting the original components with our proposed ones leads to steady enhancements in underwater object detection accuracy. Our method improves AP, $AP_{50}$, and $AP_{75}$ on the RUOD dataset by 1.8%, 1.1%, and 2.2%, respectively, and it improves them on the DUO dataset by 1.5%, 0.2%, 1.6% compared to the base model gelans [35]. It is worth noting that while the performance gains are consistent, conducting several-fold cross-validation using the proposed method would provide a more robust evaluation of the standard deviation for its performance. This would allow for a more comprehensive understanding of the robustness of the proposed method and the significance of the performance improvements. Ideally, this would be carried out for all comparisons, and a t-test should be used to determine statistical significance.

*(1) Impact of Multi-Scale Detail Amplification Module (MDAM):* To explore the contribution of our MDAM module, we replace the second layer of the backbone network with MDAM. The results in Table 5 and Table 6 clearly demonstrate that MDAM improves feature extraction. Additionally, we perform several experiments to examine how different parameter configurations affect MDAM. As shown in Figure 3, MDAM consists of three branches that use different kernel sizes and dilation rates. We test various combinations of these parameters, and the results are presented in Table 7. The *i*th branch uses $1\times k_{i}$ and $k_{i}\times 1$ convolution kernels, as well as a dilated convolution with a dilation rate of $k_{i}$. When $k_{2}$, $k_{3}$, and $k_{4}$ are set to 3, 7, and 9 and 3, 5, and 7, the performance shows little variation. However, when these values are set to combinations of 3, 5, and 9 and 5, 7, and 9, a slight decrease in detection performance occurs. Different kernel sizes and dilation rates affect the receptive field. An excessively large receptive field reduces the model’s sensitivity to certain detailed features, weakening the MDAM’s ability to capture local characteristics and fine details. In all experiments, unless specified otherwise, $k_{2}$, $k_{3}$, and $k_{4}$ are set to 3, 5, and 7.

**Figure 8:**
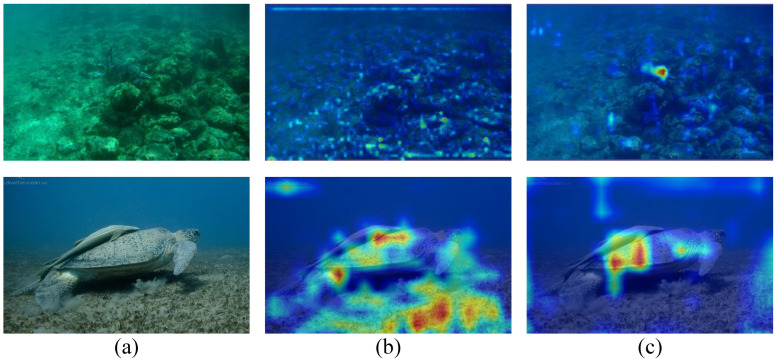
**Visualization comparison results of the heat map.** Column (**a**) presents the input image. Column (**b**) shows the heat map of the baseline, and column (**c**) shows the heat map of our SEANet.

*(2) Semantic Enhancement Feature Pyramid (SE-FPN):* In the main module CEM of the SE-FPN, in addition to introducing a foreground–background contrast mechanism, we also apply SCAM operations to the input to achieve dynamic perception. As shown in Table 5, when we replace the original feature pyramid with our SE-FPN without altering other parts of the model, our net achieves 66.1% AP, 87.7% $AP_{50}$, 72.7% $AP_{75}$, 80.8% R, 87.3% P, and 83.9% F1. On this basis, after adding the MDAM module, the complete network achieves 67.0% AP, 88.4% $AP_{50}$, 73.9% $AP_{75}$, 82.0% R, 87.6% P, and 84.7% F1. To gain a deeper and more intuitive understanding, we illustrate the heat maps of two images in Figure 8, corresponding to the feature for the first detection head. It can be observed that the baseline model fails to effectively focus on the foreground target, whereas our model enhances the distinction between foreground and background features.

### 4.5. Qualitative Comparisons

**Figure 9:**
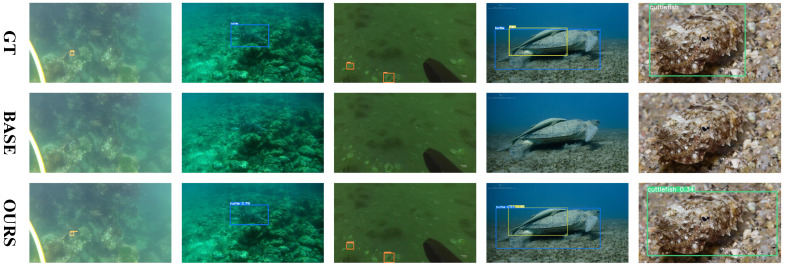
**Comparison of partial results from the RUOD dataset.** The first row shows the ground truth labels, the second row presents the baseline detection results, and the third row displays the detection results from our SEANet.

**Figure 10:**
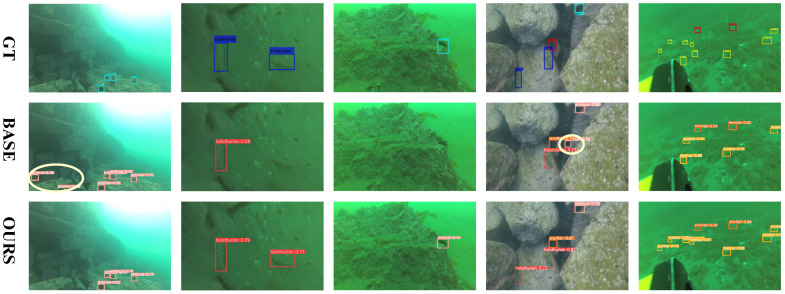
**Comparison of partial results from the URPC2021 dataset.** The first row shows the ground truth labels, the second row presents the baseline detection results, and the third row displays the detection results from our SEANet. The purple circles represent labels that are incorrectly identified as positive.

**Figure 11:**
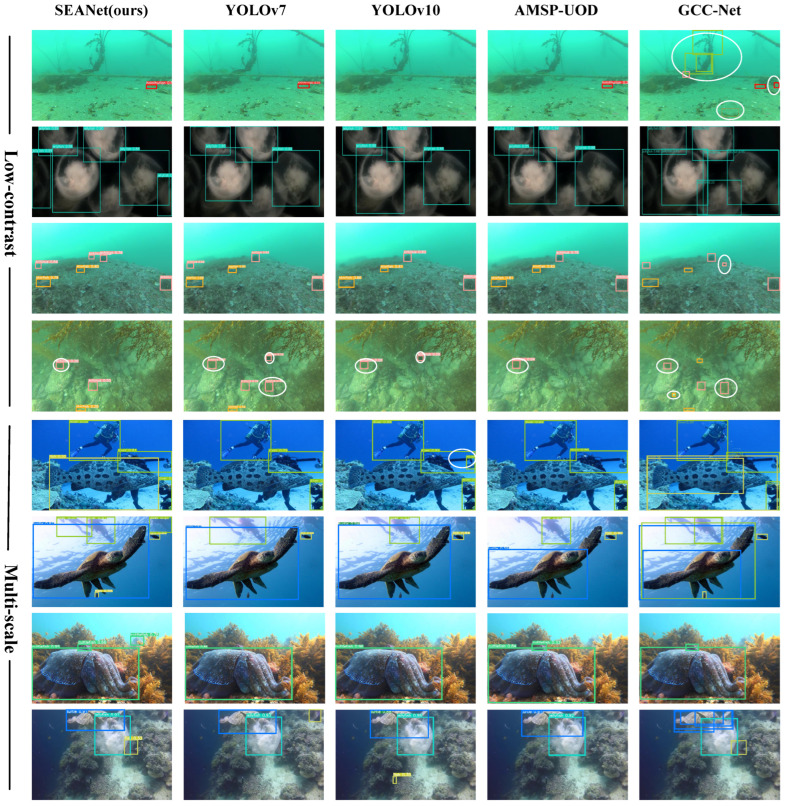
**Qualitative comparison of our method with others.** Each color of the annotation box corresponds to a specific organism, with the white circle representing a miscount. Missed detections are not marked with any special symbols in the image.

Figure 9 and Figure 10 provide a clear comparison between our method and the baseline. SEANet successfully detects several underwater organisms that closely resemble the background, which the baseline method fails to identify. Furthermore, Figure 11 shows the qualitative comparison between SEANet and other methods on the RUOD dataset. We apply the detectors to two challenging scenarios, each addressing one of the two main difficulties in Low-Contrast and Multi-Scale Underwater Object Detection (LMUOD): low contrast between objects and their backgrounds and varying object sizes. To evaluate performance, we categorize the selected images into two groups, each containing four images corresponding to these challenges. Rows 1–4 present detection results under low-contrast conditions. In these images, numerous objects (mainly holothurian, echinus, starfish, and scallop) are scattered across the seabed or rocks, complicating detection efforts. In the first row of images, a holothurian that closely resembles the surrounding background is not detected by YOLOv10. While GCC-Net can detect it, it mistakenly identifies the cluttered background as echinus and divers. In scenarios where the model’s perception is weak and many objects are present, our method still accurately detects targets. For example, in the second row, it successfully identifies the jellyfish on the left, and in the third row, it detects the distant echinus. The results from rows 5–8 demonstrate that our method excels at handling multi-scale features, effectively improving detection performance for objects of varying sizes. The fifth row features a fish and three divers, with the fish positioned at the visual center and partially obstructing the divers behind it. Our SEANet successfully detects all four targets, while YOLOv7, YOLOv10, and AMSP-UOD fail to identify the largest fish. In the sixth row of images, the bounding box of the largest turtle is approximately 399 times that of the smallest fish. Among the five detection methods, only SEANet and GCC-Net simultaneously identify both the largest and smallest targets, but GCC-Net fails to detect the diver in the upper right corner. In the seventh row, SEANet is the only method that successfully detects all three cuttlefish. Overall, Figure 11 demonstrates the great performance and competitiveness of our SEANet in LMUOD.

### 4.6. Analysis on Robustness of the Detector

Autonomous underwater vehicles (AUVs) encounter several challenges in underwater environments, including motion blur and noise. These issues hinder the accuracy of object detection, as AUVs depend on sensors and cameras to identify marine organisms in real-time. Thus, assessing the robustness of detection methods under these conditions is essential for ensuring reliable AUV performance. Based on this, we conduct a robustness analysis of our model on the RUOD dataset.

**Figure 12:**
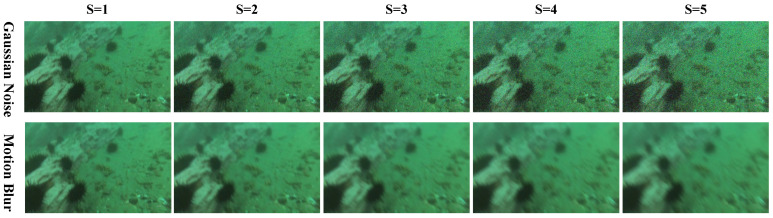
**Visualization of an image under different levels of Gaussian noise and motion blur.** The variable S denotes the severity level of the disturbance, with higher values indicating more severe degradation.

To simulate the impact of underwater noise and motion blur caused by AUVs during image capture on detection performance, we define five intensity levels, denoted as S, where higher values indicate more severe disturbances. Gaussian noise is introduced with standard deviations ranging from 10 (mild noise) to 50 (severe noise under extreme conditions). Motion blur is simulated using convolution kernels of increasing size (5 to 21), with larger kernels representing stronger blur caused by faster AUV movement. By individually evaluating the model under different levels of noise and blur, we comprehensively assess its robustness in complex underwater environments. For a more intuitive comparison across levels, a sample image is visualized in Figure 12.

**Figure 13:**
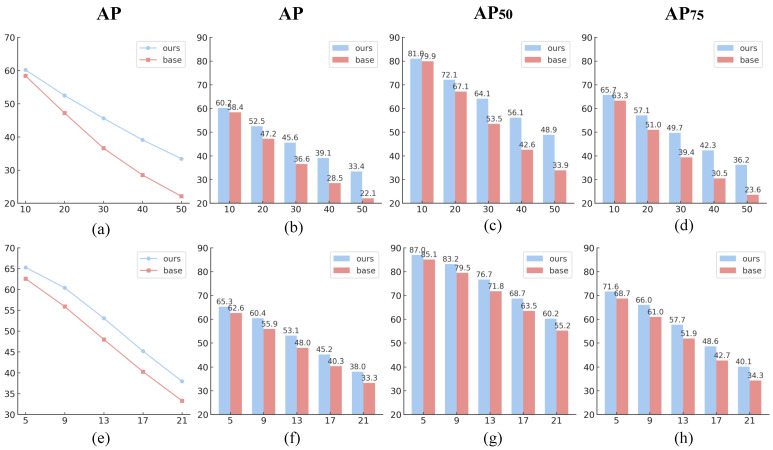
**Performance comparison under different levels of Gaussian noise and motion blur.** Subfigures (**a**–**d**) show the robustness of our method and the baseline under five levels of Gaussian noise, while (**e**–**h**) depict the robustness under five levels of motion blur. Specifically, (**a**,**e**) illustrate AP trend curves under Gaussian noise and motion blur, respectively. Subfigures (**b**–**d**) and (**f**–**h**) present bar charts comparing AP, $AP_{50}$, and $AP_{75}$ between our method and the baseline across five degradation levels (S = 1 to S = 5), corresponding to Gaussian noise standard deviations of 10, 20, 30, 40, and 50 and motion blur kernel sizes of 5, 9, 13, 17, and 21, respectively.

As illustrated in Figure 13, both our method and the baseline experience performance degradation as the severity of Gaussian noise and motion blur increases. Gaussian noise, as a pixel-level random disturbance, disrupts textures and fine details, resulting in a sharp performance drop as the standard deviation rises. In contrast, motion blur affects structural clarity without introducing randomness, leading to a more gradual decline. Nevertheless, our model consistently outperforms the baseline across all disturbance levels, with the performance gap widening as degradation intensifies. Under Gaussian noise with a standard deviation of 10, our model achieves 60.2% AP, surpassing the baseline by 1.8%. At the most extreme noise level (std = 50), our AP reaches 33.4%, while the baseline drops to 22.1%, widening the gap to 11.3%. Similar trends are observed in $AP_{50}$ and $AP_{75}$: the gap in $AP_{50}$ increases from 1.1% (81.0% vs. 79.9%) to 15.0% (48.9% vs. 33.9%) and from 2.4% (65.7% vs. 63.3%) to 12.6% (36.2% vs. 23.6%) in $AP_{75}$. Under motion blur, a more gradual degradation pattern is observed. At the mildest level (kernel size = 5), our method achieves 65.3% AP, outperforming the baseline by 2.7%. As blur intensity increases to a kernel size of 21, the gap slightly widens, with our method maintaining 38.0% AP compared to the baseline’s 33.3%, yielding a margin of 4.7%.

These results demonstrate that our model exhibits superior robustness against various types of visual degradation, maintaining relatively stable performance even under severe interference. This confirms its practical effectiveness and reliability in real-world underwater environments characterized by complex disturbances.

## 5. Discussion

In underwater object detection, addressing challenges like low-contrast and multi-scale variations is essential due to the inherent complexity of the underwater environment and the limited computational resources available on AUVs. Although lightweight models have attracted attention for their efficiency, they often struggle to capture fine-grained and multi-scale features effectively. In this paper, we propose SEANet, which not only surpasses existing methods in detection accuracy but also achieves an excellent balance between performance and computational efficiency. Notably, SEANet delivers strong detection results while maintaining a relatively low parameter count, highlighting its suitability for real-world deployment in resource-constrained underwater scenarios.

Although SEANet performs excellently, real-time applications may still face challenges related to inference speed, particularly in complex environments. Future work could explore additional optimization methods, such as model pruning or incorporating more efficient components, to further reduce computational costs. Additionally, the signal-to-noise ratio (SNR) remains a significant challenge in underwater imaging, and future research could focus on developing advanced noise reduction techniques to improve SNR, thereby further enhancing the model’s sensitivity and detection accuracy. Furthermore, we recognize that motion information in video plays a crucial role in object recognition. Therefore, future research could explore how to effectively integrate video data, leveraging motion cues to improve target detection performance in dynamic environments.

It is also important to note that different architectural and algorithmic solutions can implement the same mathematical computation, as Marr proposed in the 1980s. The choice of architecture and algorithm can significantly influence the information used and processed by the model. In this work, we have focused on presenting SEANet at the architectural/algorithmic level, but it is equally important to consider the underlying mathematical principles that guide the design of these components. Future research could delve deeper into the mathematical theory behind SEANet, exploring how different information is utilized and processed at each stage of the network. This would provide a more comprehensive understanding of why SEANet performs well and how it compares to other methods in terms of mathematical theory.

## 6. Conclusions

Underwater object detection faces new challenges compared to generic object detection due to the adeptness of aquatic organisms at camouflage, low contrast with the environment, and significant size differences between targets. In this work, we propose a Semantic Enhancement and Amplification Network (SEANet) for underwater object detection to address the challenges mentioned above. Firstly, we propose a Multi-Scale Detail Amplification Module (MDAM) to extract features that are difficult to recognize. MDAM enables a larger receptive field to expand the target area and improves the perception of detailed information. Second, we build a Semantic Enhancement Feature Pyramid (SE-FPN) to help the model effectively fuse feature information from different layers. SE-FPN leverages CEM to separate channels that emphasize low-contrast biological features from those representing background information, converting them into two distinct vectors, which aids in adaptive contrast learning. This approach enables the model to place greater emphasis on target features and effectively learn to distinguish targets that are difficult to identify in complex underwater environments. Experiments on three public underwater datasets show that SEANet surpasses other leading detectors in underwater object detection. Ablation studies validate the effectiveness of the proposed modules. We believe that our findings will offer valuable contributions to the field of underwater object detection.

## Figures and Tables

**Table 1:** The parameters used to analyze the experiments.

Type	Setting	Type	Setting
Image size	640	Momentum	0.937
Batch size	16	Weight decay	0.0005
Optimizer	SGD	Initial learning rate	0.01
Epochs	300	Seed	0

**Table 2:** Performance comparison between SEANet and other object detection methods on the RUOD dataset. The highest performance is indicated in bold, and the second highest is indicated with underlining.

Methods	Year	AP ↑	${\mathrm{AP}}_{50}↑$	${\mathrm{AP}}_{75}↑$	R ↑	P ↑	F1 ↑
**Generic Object Detector:**							
SSD [41]	2016	43.4	73.4	45.4	70.3	71.2	70.7
Faster-RCNN [42]	2016	52.8	81.8	57.5	78.7	79.4	79.0
Cascade-RCNN [43]	2018	54.8	81.1	59.7	78.5	79.2	78.8
FreeAnchor [44]	2019	55.0	82.4	59.8	79.2	80.1	79.6
NAS-FPN [45]	2019	51.4	78.9	55.2	75.7	76.6	76.1
Libra-RCNN [28]	2019	54.8	82.8	60.5	79.8	80.6	80.2
RepPoints [46]	2019	55.4	83.7	60.4	80.7	81.6	81.1
Guided-Anchoring [47]	2019	56.7	84.2	62.0	81.2	81.9	81.5
ATSS [48]	2020	52.9	80.3	56.9	77.5	78.1	77.8
Dynamic-RCNN [49]	2020	54.4	81.3	60.3	78.2	79.1	78.6
FoveaBox [50]	2020	52.1	81.4	56.0	78.2	79.0	78.6
YOLOF [51]	2021	50.1	80.0	53.8	77.0	77.9	77.4
Detecors [38]	2021	57.8	83.6	63.6	80.1	81.0	80.5
YOLOv7 [39]	2022	64.6	88.0	71.2	**82.9**	86.2	84.5
YOLOv8s ^1^	2023	63.6	86.3	69.9	80.2	86.5	83.2
YOLOv10s [40]	2024	62.8	86.2	68.9	79.9	87.1	83.3
**Underwater Object Detector:**							
RFTM [52]	2023	53.3	80.2	57.7	-	-	-
AMSP-UOD [22]	2024	65.2	86.1	72.5	79.4	86.6	82.8
DJL-Net [53]	2024	57.5	83.7	62.5	-	-	-
Dynamic YOLO [21]	2024	63.7	87.0	69.8	81.1	86.1	83.5
GCC-Net [8]	2024	59.4	85.6	65.6	81.2	86.0	83.5
SEANet(ours)	-	**67.0**	**88.4**	**73.9**	82.0	**87.6**	**84.7**

^1^ https://github.com/ultralytics/ultralytics/tree/main/ultralytics/cfg/models/v8 (accessed on 1 August 2024).

**Table 3:** Performance comparison between SEANet and other object detection methods on the URPC2021 dataset.

Methods	Year	URPC			URPC Categories AP50			
		**AP** ↑	${\mathrm{AP}}_{50}↑$	${\mathrm{AP}}_{75}↑$	**Ho** ↑	**Ec** ↑	**St** ↑	**Sc** ↑
FoveaBox [50]	2020	45.6	81.7	45.9	74.9	90.7	87.8	73.6
Dynamic-RCNN [49]	2020	45.4	78.8	47.6	71.7	87.8	85.8	70.0
Double-Head R-CNN [54]	2020	45.8	81.0	47.3	74.1	90.0	87.2	72.5
Detectors [38]	2021	46.2	80.4	49.1	73.5	89.0	86.3	72.6
TOOD [55]	2021	47.8	82.3	51.0	76.4	88.3	88.4	76.1
YOLOX [56]	2021	43.8	80.2	43.3	69.3	90.0	86.3	75.0
YOLOv7 [39]	2022	49.7	85.2	53.1	**78.4**	**92.3**	90.1	79.9
YOLOv8s	2023	50.9	84.4	55.7	76.6	90.8	89.8	80.6
AMSP-UOD [22]	2024	49.8	82.8	54.6	72.7	90.3	87.4	80.8
YOLOv10m [40]	2024	51.2	84.8	56.5	76.3	91.2	89.7	81.8
Dynamic YOLO [21]	2024	52.7	**85.5**	59.8	77.5	92.0	90.2	82.1
GCC-Net [8]	2024	49.4	83.8	53.2	78.0	89.0	89.5	78.6
SEANet (ours)	-	**53.0**	**85.5**	**60.3**	77.8	91.5	**90.3**	**82.3**

Bold denotes the best performance in each column, while underlined values represent the second-best.

**Table 4:** Performance comparison between SEANet and other detection methods on the DUO dataset.

Methods	Params	DUO			DUO Categories AP			
		**AP** ↑	${\mathrm{AP}}_{50}↑$	${\mathrm{AP}}_{75}↑$	**Ho** ↑	**Ec** ↑	**Sc** ↑	**St** ↑
**Generic Object Detector:**								
Faster R-CNN [42]	41.17	61.3	81.9	69.5	61.4	70.4	41.9	71.4
Cascade R-CNN [43]	68.94	61.2	82.1	69.2	61.9	69.0	41.9	72.0
DetectoRS [38]	123.23	64.8	83.5	72.4	65.8	73.5	45.7	74.3
GFL [57]	32.04	65.5	83.7	71.9	64.3	74.2	47.5	75.9
YOLOv7 [39]	37.25	66.3	85.8	73.9	66.3	73.7	50.8	74.5
YOLO11m [58]	20.1	71.3	86.8	78.4	70.7	77.9	56.8	**79.7**
**Underwater Object Detector:**								
RoIMix [13]	68.94	61.9	81.3	69.9	63.0	70.7	41.7	72.4
Boosting R-CNN [14]	45.95	63.5	78.5	71.1	63.8	69.0	46.8	74.5
ERL-Net [59]	218.83	64.9	82.4	73.2	67.2	71.0	46.5	74.8
GCC-Net [8]	38.31	69.1	**87.8**	76.3	68.2	75.2	56.3	76.7
RFTM [52]	75.58	60.1	79.4	68.1	-	-	-	-
AMSP-UOD [22]	10.36	68.5	84.8	76.7	65.6	**78.0**	53.3	77.3
DJL-Net [53]	58.48	65.6	84.2	73.0	-	-	-	-
SEANet(ours)	24.9	**71.5**	**87.8**	**79.1**	**71.4**	77.6	**57.8**	79.0

**Table 5:** Ablation study on the impact of each module’s effectiveness on the RUOD dataset.

Methods	AP ↑	${\mathrm{AP}}_{50}↑$	${\mathrm{AP}}_{75}↑$	R ↑	P ↑	F1 ↑
base	65.2	87.3	71.7	80.2	86.4	83.2
base + MDAM	65.8	87.6	72.2	80.8	86.7	83.6
base + SE-FPN(all)	66.1	87.7	72.7	80.8	87.3	83.9
base + MDAM + SE-FPN (w/o SCAM)	66.7	88.0	73.3	81.6	87.4	84.4
base + MDAM + SE-FPN (w/o CEM)	66.4	88.1	73.0	81.0	87.5	84.1
base + MDAM + SE-FPN (all)	67.0	88.4	73.9	82.0	87.6	84.7
base + MDAM + BiFPN	65.8	87.8	72.5	81	86.8	83.8

**Table 6:** Ablation study on the impact of each module’s effectiveness on the DUO dataset.

Methods	AP ↑	${\mathrm{AP}}_{50}↑$	${\mathrm{AP}}_{75}↑$	Ho ↑	Ec ↑	Sc ↑	St ↑
base	70.0	87.6	77.5	69.5	77.0	55.5	78.1
base + MDAM	70.9	87.9	78.6	71.1	77.5	56.4	78.8
base + SE-FPN (all)	71.0	87.5	78.7	70.9	77.4	56.7	78.9
base + MDAM + SE-FPN (w/o SCAM)	71.1	87.5	78.8	71.0	77.5	57.4	78.5
base + MDAM + SE-FPN (all)	71.5	87.8	79.1	71.4	77.6	57.8	79.0

**Table 7:** Ablation study on the impact of different parameters in MDAM.

Branch2	Branch3	Branch4	AP ↑	${\mathrm{AP}}_{50}↑$	${\mathrm{AP}}_{75}↑$	R ↑	P ↑	F1 ↑
3	7	9	67.0	88.3	74.0	82.5	86.4	84.4
3	5	7	67.0	88.4	73.9	82.0	87.6	84.7
3	5	9	66.6	88.1	73.1	82.0	87.1	84.5
5	7	9	66.7	87.9	73.5	81.5	87.3	84.3

## Data Availability

The data that support the findings of this study are publicly available and can be accessed from the RUOD https://github.com/dlut-dimt/RUOD, DUO https://github.com/chongweiliu/DUO and URPC2021 https://pan.baidu.com/s/1YVIpvYPg8jbXOUE0LFqcJw?pwd=uvwm (accessed on 1 March 2025). For further details, please refer to the dataset citations or contact the corresponding author.

## Abbreviations

The following abbreviations are used in this manuscript:

SEANet	Semantic Enhancement and Amplification Network;
SE-FPN	Semantic Enhancement Feature Pyramid;
MDAM	Multi-Scale Detail Amplification Module;
CEM	Contrast Enhancement Module;
FBC	Fore-Background Contrast Attention;
UOD	Underwater object detection;
AP	Average Precision;
AUVs	Autonomous underwater vehicles
